# Renal impairment in transcatheter aortic valve implantation: incidence, predictors, and prognostic significance

**DOI:** 10.1186/s12872-025-04982-4

**Published:** 2025-07-18

**Authors:** Abdelrahman Ahmed Abdelrahman, Mahmoud Baraka, Nabil Farag, Ahmed E. Mostafa, Diaa Kamal

**Affiliations:** Cardiology Department, Faculty of Medicine, Ain Shams University, Cairo, 11591 Egypt

**Keywords:** TAVI, Renal impairment, Contrast volume, Ejection fraction, Ad hoc revascularization

## Abstract

**Background:**

Renal impairment is a recognized complication of transcatheter aortic valve implantation (TAVI), impacting morbidity and mortality. Understanding its incidence, predictors, and prognostic implications is essential to optimizing patient outcomes.

**Purpose:**

To determine the incidence, predictors, and prognostic significance of renal impairment in patients undergoing TAVI.

**Methods:**

This prospective observational study was conducted on 147 patients, with 144 completing the study. Patients with severe symptomatic aortic stenosis (aortic valve area (AVA) < 1 cm²) were included. Clinical, echocardiographic, and procedural parameters were analyzed to identify predictors of post-TAVI renal impairment, defined per Valve Academic Research Consortium (VARC)-2 AKIN criteria.

**Results:**

Renal impairment occurred in 13.9% of patients post-TAVI. Compared to those without impairment, affected patients more frequently underwent ad-hoc revascularization (90% vs. 21.3%, *P* < 0.001), received higher contrast volume (median 200 mL vs. 130 mL, *P* < 0.001), and had longer procedures (82.5 ± 29 vs. 60.9 ± 28.3 min, *P* = 0.002). They also exhibited lower post-procedural ejection fraction (EF) (47.4% ± 9.7% vs. 59.8% ± 9.7%, *P* < 0.001) and higher incidence of regional wall motion abnormalities (60% vs. 12.3%, *P* < 0.001). Multivariate analysis identified ad-hoc revascularization (OR = 448.7, 95% CI: 17.09–11778.5, *P* < 0.001), lower EF (OR = 0.87, 95% CI: 0.79–0.97, *P* = 0.009), and contrast volume (OR = 0.98, 95% CI: 0.96–1.00, *P* = 0.045) as independent predictors.

**Conclusions:**

Renal impairment post-TAVI is multifactorial, with contrast volume, ad hoc revascularization, and reduced EF as key independent predictors. Minimizing contrast use and optimizing procedural strategies may mitigate renal risk and improve patient outcomes.

## Introduction

Aortic valvular disease is a prevalent condition, particularly among elderly individuals with multiple comorbidities. Among its various forms, senile calcific aortic stenosis (AS) is the most common, characterized by progressive valve calcification and narrowing, leading to left ventricular outflow obstruction [1]. Despite advancements in medical therapies, no pharmacological treatment effectively alters the disease course once symptoms appear or left ventricular dysfunction develops. As a result, surgical aortic valve replacement (SAVR) remains the standard treatment for severe AS, offering significant symptom relief and survival benefits [2]. AS may present insidiously or acutely, with symptoms ranging from exertional dyspnea, syncope, and angina to acute heart failure or cardiogenic shock in advanced cases [3]. In such cases, prompt diagnosis and intervention are critical.

However, because aortic stenosis predominantly affects older adults, many patients present with significant comorbidities, which may increase surgical risk or render them ineligible for SAVR [4]. In such cases, minimally invasive alternatives have gained attention. One such approach is transcatheter aortic valve implantation (TAVI), which provides a less invasive means of valve replacement, making it a viable option for patients deemed high-risk or inoperable for conventional surgery [5].

While percutaneous balloon aortic valvuloplasty was initially considered an option, its limited durability has restricted its role in the management of calcific AS [6]. In contrast, TAVI has demonstrated remarkable efficacy in treating severe aortic stenosis in patients at high surgical risk or those deemed inoperable [7]. Since Alain Cribier performed the first-in-human implantation in 2002 [8], TAVI has rapidly evolved, becoming a leading alternative to SAVR with continuous advancements in device technology and procedural techniques [9].

Despite the clinical benefits of TAVI, growing experience has revealed several intra- and post-procedural complications, one of the most significant being renal impairment [10]. Post-procedural renal dysfunction can negatively impact patient outcomes, increasing morbidity and mortality. Given the aging population undergoing TAVI and their higher baseline renal vulnerability, understanding the incidence, predictors, and prognostic implications of renal impairment in these patients is crucial [11].

This study aims to determine the incidence, predictors, and prognostic significance of renal impairment in patients undergoing TAVI.

## Patients and methods

### Design and population

This prospective observational study was conducted at Ain Shams University Hospitals from May 2022 to May 2024. A total of 147 patients were initially enrolled; however, three patients were excluded due to in-hospital mortality, resulting in a final study cohort of 144 patients. The excluded cases included one patient who succumbed to stroke, one due to hospital-acquired pneumonia, and one due to cardiac perforation.

The study protocol was approved by the Scientific and Ethical Committee of the Faculty of Medicine, Ain Shams University. All patients were informed about the study and provided verbal or written consent.

### Eligibility criteria

Patients were eligible for inclusion if they had severe symptomatic aortic stenosis, defined as an aortic valve area (AVA) < 1 cm² or < 0.6 cm²/m², with or without accompanying aortic regurgitation. Additionally, patients were required to have an aortic valve annulus diameter between 18 mm and 29 mm, ensuring suitability for TAVI.

Patients were excluded if they had an aortic valve annulus size incompatible with TAVI, severe renal impairment (chronic kidney disease (CKD) stage 4 or 5, eGFR < 30 mL/min/1.73 m²), left ventricular or aortic thrombi, advanced disease with a life expectancy < 1 year, active sepsis or endocarditis, or contraindications to study medications.

### All patients were subjected to the following

#### Pre-procedural parameters

Were assessed to identify potential predictors of post-procedural renal impairment. Clinical parameters included age, gender, and common cardiac risk factors such as diabetes mellitus, hypertension, smoking, ischemic heart disease, previous cerebrovascular stroke (CVS), and CKD stage 1–3. Additional risk factors, including bronchial asthma, anemia, chronic obstructive pulmonary disease (COPD), and prior mitral valve replacement (MVR), were also analyzed.

#### Procedural parameters

Among the 144 patients, 115 received the Medtronic Evolut R valve [12], 25 received the Boston Accurate Neo valve, and 4 received the balloon-expandable Edwards Sapien 3 valve [13]. Valve size selection was based on aortic annulus perimeter and area measurements obtained via CT imaging, following manufacturer recommendations [14]. The transfemoral approach was used in most cases, while only 3 patients required a subclavian approach due to complex anatomy and severe peripheral arterial disease. Vascular closure was performed using either ProGlide or open surgical access. Sedation methods varied, with most patients undergoing conscious sedation, while 5 received local anesthesia and 32 underwent full sedation.

Procedure duration ranged from 20 to 120 min, depending on anatomical complexity, with contrast volume ranging from 15 to 350 mL. Predilatation was performed in 78 patients, particularly in cases of rheumatic or bicuspid aortic valves, severe calcification, or when using the Sapien 3 or Accurate Neo valve. Postdilatation was required in 35 patients due to residual gradients, significant paravalvular leak (PVL), or transvalvular regurgitation, while both predilatation and postdilatation were performed in 25 patients. Additionally, ad hoc percutaneous coronary intervention (PCI) was performed in 49 patients to treat focal simple lesions based on multislice computed tomography (MSCT) coronary analysis.

Ejection fraction (EF) and resting segmental wall motion abnormalities (RSWMA) were evaluated using pre-procedural transthoracic echocardiography in all patients. AVA using the continuity equation, indexed AVA (AVAi), and mean and peak transvalvular gradients were also evaluated. All patients underwent ECG-gated CT aortography (TAVI protocol) using GE Healthcare, with measurements analyzed by two expert operators using OsiriX MD v.9.0 software following standardized imaging recommendations [14]. CT parameters included aortic annulus mean diameter, perimeter, and area indexed to body surface area (BSA), as well as coronary ostia height (Left main coronary artery (LMCA) and Right coronary artery (RCA)) with indexed values to BSA (LMCAi, RCAi). Using coronal views, the distance between the annulus and coronary ostia and the length of coronary leaflets were assessed to guide procedural planning and minimize the risk of coronary obstruction, following best practices in MSCT evaluation for transcatheter aortic valve replacement [15].

#### Aortic valve and septal calcification assessment

Aortic valve calcification was graded based on established criteria [15, 16] into four categories: Grade 1 (no calcification), Grade 2 (mild calcification with small isolated spots), Grade 3 (moderate calcification with multiple larger spots), and Grade 4 (heavy calcification involving the entire circumference). Basal ventricular septum calcification was assessed using coronal CT imaging [17] and categorized as 0 (no calcification) or 1 (presence of calcification). Additionally, the membranous septum (MS) length was measured as the distance from the aortic valve to the crest of the muscular interventricular septum, with indexed values to BSA for procedural planning.

#### Device description and procedural details

The CoreValve system consists of a trileaflet bioprosthetic porcine pericardial tissue valve mounted on a self-expanding nitinol stent. The Evolut R device, equipped with the EnVeo R delivery system and a built-in InLine sheath (14-Fr equivalent), was primarily used, along with the third-generation CoreValve (18-Fr) based on availability. Additionally, the Edwards Sapien XT valve, a cobalt-chromium balloon-expandable stent with an integrated trileaflet bovine tissue valve, was used with the NovaFlex catheter and 16–20 Fr eSheath. Vascular access was obtained percutaneously through the common femoral artery, with or without pre-planned surgical cutdown, depending on the availability of vascular closure devices. The procedure was performed under local anesthesia with mild sedation/analgesia or general anesthesia as required.

At the start of the procedure, a temporary transvenous pacemaker was positioned in the right ventricle via transjugular or transfemoral access and remained in place for at least 24 h post-TAVI, ensuring there were no signs of AV block or bradycardia before removal. Balloon valvuloplasty under rapid pacing was performed selectively before device placement. The valve was then deployed retrogradely over a stiff guidewire placed in the left ventricle under fluoroscopic guidance, with post-implantation balloon valvuloplasty performed in some cases. Electrocardiographic monitoring was continuous throughout the procedure, and postoperatively, patients were transferred to the intensive care unit (ICU) for an average of three days for continuous monitoring of heart rhythm and vital signs.

#### Post-interventional management

Patients underwent clinical follow-up, electrocardiography (ECG), and transthoracic echocardiography (TTE) post-procedure, at hospital discharge, and 30 days after device implantation. Antiplatelet and antithrombotic therapy included acetylsalicylic acid (75 mg/day) initiated pre-procedure and continued indefinitely, along with clopidogrel (300 mg loading dose), followed by 75 mg daily for life. During the procedure, a weight-adjusted heparin bolus was administered to maintain an activated clotting time (ACT) of 250–300 s. Renal function was assessed post-TAVI according to VARC-2 recommendations, with acute kidney injury (AKI) diagnosed using AKIN criteria based on Kidney Disease: Improving Global Outcomes (KDIGO) guidelines [18].

Hydration protocols were not standardized across all patients and were administered based on operator discretion. Data on the use of nephrotoxic medications (e.g., NSAIDs, aminoglycosides) were not collected prospectively.

### Statistical methods

Data were collected, coded, and analyzed using SPSS version 27. Descriptive statistics were applied based on data type: parametric numerical data were expressed as mean ± standard deviation (SD) and range, while non-parametric numerical data were reported as median and interquartile range (IQR). Categorical variables were presented as frequency and percentage. For analytical statistics, a Student’s t-test was used to compare means between two groups, while the Mann-Whitney U test was applied for non-parametric variables. The Chi-square test assessed relationships between qualitative variables, with Fisher’s exact test used when expected counts were less than 5 in more than 20% of cells. Logistic regression analysis was performed to predict the presence or absence of an outcome based on independent variables, particularly for categorical outcomes. Statistical significance was set at *P* < 0.05, while *P* > 0.05 was considered non-significant.

## Results

Patients with renal impairment had a slightly higher mean age compared to those without impairment (73.95 ± 5.09 vs. 72.21 ± 7 years, *P* = 0.29), though the difference was not statistically significant. COPD was significantly more prevalent in the renal impairment group (35% vs. 6.56%, *P* < 0.001). Other variables including gender (female: 80% vs. 63.11%, *P* = 0.141), smoking (15% vs. 6.56%, *P* = 0.187), hypertension (65% vs. 63.93%, *P* = 0.927), diabetes mellitus (20% vs. 42.62%, *P* = 0.082), ischemic heart disease (5% vs. 8.2%, *P* = 1.00), atrial fibrillation (0% vs. 13.11%, *P* = 0.128), CKD (0% vs. 4.1%, *P* = 1.00), and other comorbidities did not show significant differences between groups (*P* > 0.05) Table 1.

**Table 1:** Demographic data and past medical history between study groups

		Total	Occurrence of Renal Impairment		*P*-value
			No (*N* = 122)	Yes (*N* = 20)	
Age		72.46 ± 6.78	72.21 ± 7	73.95 ± 5.09	0.29
Gender	Male	49 (34.51%)	45 (36.89%)	4 (20%)	0.141
	Female	93 (65.49%)	77 (63.11%)	16 (80%)	
Smoker		11 (7.75%)	8 (6.56%)	3 (15%)	0.187
HTN		91 (64.08%)	78 (63.93%)	13 (65%)	0.927
DM		56 (39.44%)	52 (42.62%)	4 (20%)	0.082
IHD		11 (7.75%)	10 (8.2%)	1 (5%)	1.00
PAD		5 (3.52%)	5 (4.1%)	0 (0%)	1.00
Afib		16 (11.27%)	16 (13.11%)	0 (0%)	0.128
CKD		5 (3.52%)	5 (4.1%)	0 (0%)	1.00
CLD		3 (2.11%)	3 (2.46%)	0 (0%)	1.00
BA		8 (5.63%)	8 (6.56%)	0 (0%)	0.601
Anemia		9 (6.34%)	9 (7.38%)	0 (0%)	0.360
MVR		6 (4.23%)	6 (4.92%)	0 (0%)	0.595
COPD		15 (10.56%)	8 (6.56%)	7 (35%)	< 0.001*
Thyroid dysfunction		12 (8.45%)	10 (8.2%)	2 (10%)	0.677

Data were presented as Mean ± SD, *n* (%)

*HTN* Hypertension, *DM* Diabetes Mellitus, *IHD* Ischemic Heart Disease, *PAD* Peripheral Arterial Disease, *Afib* Atrial Fibrillation, *CKD* Chronic Kidney Disease, *CLD* Chronic Liver Disease, *BA* Bronchial Asthma, *MVR* Mitral Valve Replacement, *COPD* Chronic Obstructive Pulmonary Disease

*Statistically significant *p*-value as *p* < 0.05

Patients with renal impairment were significantly more likely to undergo ad-hoc revascularization (90% vs. 21.31%, *P* < 0.001), predilatation (70% vs. 45.9%, *P* = 0.046), both pre- and postdilatation (35% vs. 10.66%, *P* = 0.004), and received a higher median contrast volume (200 [150–250] ml vs. 130 [100–200] ml, *P* < 0.001). Additionally, they had a longer mean procedure duration (82.5 ± 29 min vs. 60.9 ± 28.26 min, *P* = 0.002). Other variables, including pre-procedural creatinine (*P* = 0.7), access type (*P* = 0.582), sedation method (*P* = 0.313), valve type (*P* = 0.101), and pacing rate (*P* = 0.706), did not differ significantly between groups Table 2.

**Table 2:** Pre and Intra-operative data between study groups

		Total	Occurrence of Renal Impairment		*P*-value
			No (*N* = 122)	Yes (*N* = 20)	
Pre creatinine		1 (0.89–1.2)	1 (0.85–1.2)	0.97 (0.9–1.1)	0.7
Adhoc Revasc	No	98 (69.01%)	96 (78.69%)	2 (10%)	< 0.001*
	Yes	44 (30.99%)	26 (21.31%)	18 (90%)	
Access	Proglide	71 (50%)	63 (51.64%)	8 (40%)	0.582
	Open (Subclavian artery)	3 (2.11%)	3 (2.46%)	0 (0%)	
	Open (Femoral)	68 (47.89%)	56 (45.9%)	12 (60%)	
Sedation	Conscious sedation	31 (21.83%)	29 (23.77%)	2 (10%)	0.313
	Full	106 (74.65%)	88 (72.13%)	18 (90%)	
	Local anethesia	5 (3.52%)	5 (4.1%)	0 (0%)	
Predilataion (Use ballon)	No	72 (50.7%)	66 (54.1%)	6 (30%)	0.046*
	Yes	70 (49.3%)	56 (45.9%)	14 (70%)	
Average size of balloon used in Predilataion (mm)		22.06 ± 2.05	21.93 ± 2.03	22.57 ± 2.1	0.297
Type of Valve	Sapien 3	4 (2.82%)	2 (1.64%)	2 (10%)	0.101
	Evolut R	113 (79.58%)	97 (79.51%)	16 (80%)	
	Accurate neo 2	25 (17.61%)	23 (18.85%)	2 (10%)	
Duration of the procedure (minutes)		63.94 ± 29.25	60.9 ± 28.26	82.5 ± 29	0.002*
Postdilatation (Use ballon)	No	112 (78.87%)	99 (81.15%)	13 (65%)	0.101
	Yes	30 (21.13%)	23 (18.85%)	7 (35%)	
Postdilatation (mm)		24.17 ± 2.6	23.96 ± 2.9	24.86 ± 1.07	0.432
Both predilatation and postdilatation	No	122 (85.92%)	109 (89.34%)	13 (65%)	0.004*
	Yes	20 (14.08%)	13 (10.66%)	7 (35%)	
Amount of contrast (ml)		150 (100–200)	130 (100–200)	200 (150–250)	< 0.001*
Pacing (BPM)		153.73 ± 31.03	153.2 ± 28.81	157 ± 42.93	0.706

Data were presented as Mean ± SD, *n* (%), Mean (IQR)

*Adhoc Revasc* Adhoc Revascularization, *AS* Aortic Stenosis, *ml* Milliliters, *mm *Millimeters, *Postdilatation* Post-procedural Balloon Dilation, *Predilatation* Pre-procedural Balloon Dilation, *TAVI* Transcatheter Aortic Valve Implantation, *BPM* Beats per minute

*Statistically significant *p*-value as *p* < 0.05

Postoperative EF was significantly lower in patients with renal impairment (47.4% ± 9.7% vs. 59.8% ± 9.7%, *P* < 0.001), and regional wall motion abnormalities were more frequent (60% vs. 12.3%, *P* < 0.001). No significant differences were observed between groups in terms of overall morbidity (*P* = 0.782), readmission rates (*P* = 0.129), or improvement in functional capacity over follow-up (*P* = 0.792). Mean and peak transvalvular gradients also did not differ significantly (*P* = 0.189 and *P* = 0.413, respectively) Table 3.

**Table 3:** Post-operative & ECHO findings between study groups

		Total	Occurrence of Renal Impairment		*P*-value
			No (*N* = 122)	Yes (*N* = 20)	
Morbidity	No	127 (89.44%)	107 (87.7%)	20 (100%)	0.782
	Complete heart block & device implantation	5 (3.52%)	5 (4.1%)	0 (0%)	
	Improved EF	1 (0.7%)	1 (0.82%)	0 (0%)	
	Stroke	7 (4.93%)	7 (5.74%)	0 (0%)	
Readmission	Chest infection	2 (1.41%)	2 (1.64%)	0 (0%)	
	No	127 (89.44%)	107 (87.7%)	20 (100%)	0.129
	Yes	15 (10.56%)	15 (12.3%)	0 (0%)	
Functional Capacity	Didn’t improved	16 (11.27%)	13 (10.66%)	3 (15%)	0.792
	Improved after 1 month	48 (33.8%)	40 (32.79%)	8 (40%)	
	Improved after 3 months	52 (36.62%)	46 (37.7%)	6 (30%)	
	Improved after 6 months	26 (18.31%)	23 (18.85%)	3 (15%)	
EF%		58% ± 10.6%	59.8% ± 9.7%	47.4% ± 9.7%	< 0.001*
RSWMA	No	115 (80.99%)	107 (87.7%)	8 (40%)	< 0.001*
	Yes	27 (19.01%)	15 (12.3%)	12 (60%)	
Mean gradient (mmHg)		5 (3–7)	5 (3–7)	5 (5–6)	0.189
Peak gradient (mmHg)		10 (8–14)	10 (8–14)	10 (10–10)	0.413

Data were presented as Mean ± SD, *n* (%), Mean (IQR)

*EF* Ejection Fraction, *mmHg* Millimeters of Mercury, *RSWMA* Regional Wall Motion Abnormality

*Statistically significant *p*-value as *p* < 0.05

**Fig. 1:**
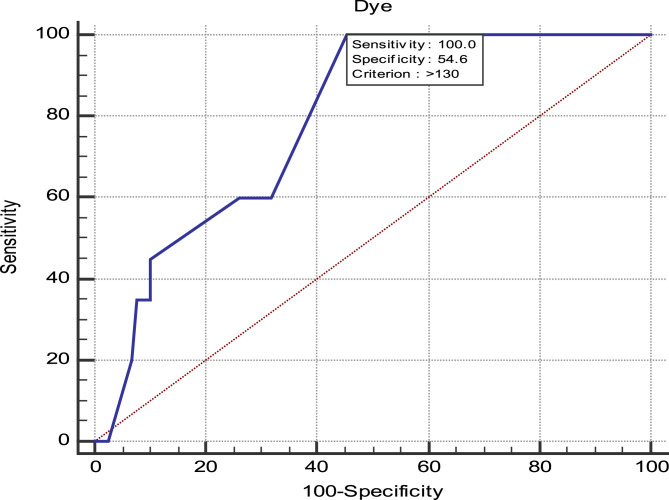
ROC curve for dye volume in predicting renal impairment

The amount of dye used demonstrated strong predictive ability for renal impairment, with an area under the curve (AUC) of 0.788 (95% CI: 0.704–0.872, p-value < 0.001). A cutoff of > 130 mL yielded 100% sensitivity, 54.62% specificity, a positive predictive value (PPV) of 27%, and a negative predictive value (NPV) of 100% Fig. 1.

In univariate analysis, ad-hoc revascularization (OR = 33.23, 95% CI: 7.24–152.51, *P* < 0.001), procedure duration (OR = 1.02, 95% CI: 1.01–1.04, *P* = 0.003), contrast volume (OR = 1.01, 95% CI: 1.01–1.02, *P* < 0.001), reduced EF (OR = 0.89, 95% CI: 0.85–0.94, *P* < 0.001), and presence of RSWMA (OR = 10.7, 95% CI: 3.76–30.43, *P* < 0.001) were significantly associated with renal impairment. Table 4.

**Table 4:** Regression analysis model to predict occurrence of renal impairment

	Univariate analysis		Multivariate analysis	
	OR (95% CI)	*P*-value	OR (95% CI)	*P*-value
Adhoc Revasc	33.23 (7.24–152.51)	< 0.001*	448.7 (17.09–11778.52)	< 0.001*
Predilataion (Use ballon)	2.75 (0.99–7.63)	0.052	1.33 (0.15–11.83)	0.801
Duration (minutes)	1.02 (1.01–1.04)	0.003*	1.03 (1–1.05)	0.053
Amount of contrast (ml)	1.01 (1.01–1.02)	< 0.001*	0.98 (0.96–1)	0.045*
EF%	0.89 (0.85–0.94)	< 0.001*	0.87 (0.79–0.97)	0.009*
RSWMA	10.7 (3.76–30.43)	< 0.001*	1.84 (0.1–34.13)	0.682

*EF%* Ejection Fraction, *OR* Odds Ratio, *CI* Confidence Interval, *RSWMA* Regional Wall Motion Abnormalities, *TAVI* Transcatheter Aortic Valve Implantation

*Statistically significant *p*-value as *p* < 0.05

In multivariate analysis, ad-hoc revascularization remained a strong independent predictor (OR = 448.7, 95% CI: 17.09–11778.52, *P* < 0.001), along with lower EF (OR = 0.87, 95% CI: 0.79–0.97, *P* = 0.009) and contrast volume (OR = 0.98, 95% CI: 0.96–1.00, *P* = 0.045). Predilatation (*P* = 0.801), procedure duration (*P* = 0.053), and RSWMA (*P* = 0.682) did not retain statistical significance Table 4.

## Discussion

Severe AS is a prevalent heart valve disease, particularly among the elderly, with SAVR being the standard treatment [19]. However, TAVI has emerged as a less invasive and effective alternative, especially for patients at high surgical risk, advanced age, or with comorbidities [20]. Recent trials have demonstrated that TAVI is not inferior to SAVR in intermediate-risk patients, as shown in the SURTAVI trial. In contrast, the PARTNER 3 and Evolut Low Risk trials have indicated its non-inferiority or even superiority in low-risk patients [21, 22]. This study aimed to determine the incidence, predictors, and prognostic value of renal impairment in patients undergoing TAVI.

Renal impairment is a common complication following TAVI, attributed to hemodynamic instability during implantation and contrast agent exposure. Reported incidence varies widely, ranging from 8.3 to 58%, likely due to differences in study populations and AKI definitions [23]. A multicenter study reported an incidence of 20.7% [24]. In our study, using the updated Valve Academic Research Consortium (VARC)−2 criteria, the incidence of AKI was 15.28% [25].

Our study identified contrast volume, ad hoc revascularization, and low EF as the three main predictors of renal impairment after TAVI. Among these, contrast volume emerged as an independent predictor, emphasizing the role of contrast-induced nephrotoxicity in post-TAVI renal dysfunction.

The impact of contrast agents on AKI occurrence after TAVI remains controversial. While most studies do not establish a statistically significant correlation between contrast dose and AKI incidence [26], some evidence suggests that higher contrast volumes may contribute to AKI development. Similarly, Van Linden et al. [27] reported in a series of 270 consecutive TAAVI patients that the amount of intraoperative contrast agent influenced postoperative AKI and the need for RRT, identifying higher contrast volume as an independent risk factor for AKI. Similarly, Yamamoto et al. [28], who analyzed 415 transfemorally treated TAVI patients, demonstrated a direct correlation between increasing contrast dose and a higher prevalence of AKI.

Unlike our study, Madershahian et al. [29] reported an association between high contrast use, increased contrast induced nephropathy (CIN) incidence, and 30-day mortality in high-risk patients with pre-existing renal impairment undergoing TAAVI. However, our study excluded patients with pre-existing renal impairment, focusing instead on individuals with normal baseline renal function, followed post-TAVI to assess renal outcomes.

Building on previous studies, a major limitation remains: determining whether the relationship between increased contrast media and worse post-TAVI outcomes is associative or causative. In other words, does contrast agent itself contribute to worse outcomes, or is its increased use merely a result of procedural complexity? The pathophysiological mechanism of contrast-induced acute vasoconstriction, driven by the release of adenosine, endothelin, and other vasoconstrictors, leading to reduced renal blood flow, is well established [30]. However, the extent to which these effects impact renal function remains controversial.

In the current era of TAVI, the multimorbid condition of patients, combined with procedure-related insults such as microembolic events and bleeding complications, may outweigh the statistically observable negative effects of contrast agent administration. Understanding the pathophysiological mechanisms of AKI after TAVI is crucial, yet a clinical study like this cannot fully elucidate them. However, analyzing the association between AKI and patient- or procedure-related factors, including demographic characteristics and modifiable procedural variables, may provide valuable insights for risk assessment and procedural optimization.

Our study aligns with the FRANCE 2 registry, a French national TAVI registry that included 502 patients with low-flow, low-gradient aortic stenosis (LFLG AS) and reduced left ventricular EF (LVEF). Amabile et al. identified transapical access, pre-TAVI angina, advanced kidney failure, atrial fibrillation, and moderate to severe post-TAVI aortic regurgitation as independent predictors of outcomes in these patients [31]. Although our study did not include LFLG AS patients due to a smaller sample size (147 vs. 502 patients), we similarly found reduced EF to be an independent risk factor for renal impairment, consistent with the FRANCE 2 registry findings.

The True or Pseudo-Severe Aortic Stenosis (TOPAS) registry is a prospective multicenter study that included 287 patients with low-flow, low-gradient aortic stenosis (LFLG AS) and reduced LVEF undergoing TAVI, with 234 patients undergoing dobutamine stress echocardiography (DSE) before the procedure. The TOPAS registry identified COPD and low hemoglobin levels as independent outcome predictors post-TAVI. Additionally, substudies highlighted right ventricular dysfunction and tricuspid regurgitation as key factors limiting prognosis in patients with LFLG AS and reduced LVEF [32, 33]. Unlike our findings, this study did not establish a correlation between low EF and post-TAVI renal impairment.

The third predictor, ad hoc revascularization, was associated with a significant increase in AKI incidence among patients undergoing myocardial revascularization within one month prior to TAVI, although a recent meta-analysis [34] only indicated a trend toward significance, possibly due to a delayed effect of contrast administration. Unlike previous studies, our analysis focused solely on ad hoc revascularization, where patients with significant lesions on MSCT coronary angiography (as part of the CT TAVI protocol) or those with typical chest pain underwent revascularization. Notably, 20 out of 22 patients (90.91%) who underwent ad hoc revascularization developed AKI, suggesting that increased contrast volume during the procedure may have contributed to this outcome.

This assumption aligns with previous studies highlighting the significant role of contrast volume in AKI development. Research has shown that patients receiving a lower corrected contrast dose [29, 35] experienced better post-TAVI renal outcomes, reinforcing the importance of minimizing contrast media (CM) volume to less than 100 mL. In contrast, our study identified a cutoff of < 130 mL for reducing the risk of post-TAVI renal impairment.

It is likely that patients undergoing ad hoc revascularization had more extensive coronary artery disease, which may have contributed to lower pre-procedural EF and increased susceptibility to renal impairment due to both myocardial injury and higher contrast burden. This multifactorial interplay underscores the importance of thorough pre-procedural planning and individualized risk assessment.

Echocardiographic parameters of congestion, such as pulmonary pressures or inferior vena cava dimensions, were not systematically analyzed in relation to renal function. These measures, as highlighted by Angellotti D et al. [36], may provide valuable prognostic insights and should be considered in future studies.

Our findings highlight the importance of individualized procedural planning to mitigate renal injury. Contrast reduction alone may not be sufficient; careful pre-TAVI assessment of EF, coronary anatomy, and consideration of staging PCI and TAVI may help reduce renal stress. Additionally, prehydration, judicious use of RAAS inhibitors, and minimizing procedural complexity are key strategies clinicians should consider in high-risk patients.

The lack of standardized hydration and unrecorded use of nephrotoxic agents may have influenced renal outcomes. Prospective control of these factors is crucial to accurately evaluate modifiable risk contributors.

This study has several limitations. The small number of renal impairment cases precluded propensity score matching, limiting control for confounding factors. The modest sample size may reduce generalizability and introduce bias. Excluding patients with advanced CKD (stages 4–5) enhanced internal validity but limits applicability to the most vulnerable population. Additionally, the 30-day follow-up may not reflect the long-term prognostic impact of post-TAVI renal impairment. Future studies should include high-risk subgroups and longer follow-up to improve risk stratification and outcome assessment.

## Conclusions

Post-TAVI renal impairment is influenced by multiple factors, with contrast volume, ad hoc revascularization, and reduced EF emerging as key independent predictors. Reducing contrast exposure and refining procedural techniques may help lower the risk of renal complications and enhance patient outcomes.

## Data Availability

The data that support the findings of this study are available on request from the corresponding author.

## Abbreviations

Afib: Atrial Fibrillation
AKI: Acute Kidney Injury
AKIN: Acute Kidney Injury Network
AS: Aortic Stenosis
AVA: Aortic Valve Area
AVAi: Indexed Aortic Valve Area
AV: Atrioventricular
BPM: Beats Per Minute
BSA: Body Surface Area
CIN: Contrast Induced Nephropathy
CKD: Chronic Kidney Disease
CLD: Chronic Liver Disease
COPD: Chronic Obstructive Pulmonary Disease
CT: Computed Tomography
CVS: Cerebrovascular Stroke
DM: Diabetes Mellitus
DSE: Dobutamine Stress Echocardiography
ECG: Electrocardiography
EF: Ejection Fraction
EMS: External Masculinization Score
FRANCE 2: French National TAVI Registry
HTN: Hypertension
ICU: Intensive Care Unit
IHD: Ischemic Heart Disease
IQR: Interquartile Range
KDIGO: Kidney Disease: Improving Global Outcomes
LFLG AS: Low-Flow, Low-Gradient Aortic Stenosis
LMCA: Left Main Coronary Artery
LMCAi: Indexed Left Main Coronary Artery
LVEF: Left Ventricular Ejection Fraction
MVR: Mitral Valve Replacement
MSCT: Multislice Computed Tomography
PARTNER: Placement of Aortic Transcatheter Valves
PCI: Percutaneous Coronary Intervention
PPV: Positive Predictive Value
PVL: Paravalvular Leak
RCA: Right Coronary Artery
RCAi: Indexed Right Coronary Artery
RRT: Renal Replacement Therapy
RSWMA: Resting Segmental Wall Motion Abnormalities
SAVR: Surgical Aortic Valve Replacement
SURTAVI: Surgical or Transcatheter Aortic-Valve Replacement
TAAVI: Transapical Aortic Valve Implantation
TAVI: Transcatheter Aortic Valve Implantation
TTE: Transthoracic Echocardiography
TOPAS: True or Pseudo-Severe Aortic Stenosis
